# Red cell distribution width (RDW) as a biomarker for respiratory failure in a pediatric ICU

**DOI:** 10.1186/s12950-017-0160-9

**Published:** 2017-06-07

**Authors:** Tom Schepens, Jozef J. De Dooy, Walter Verbrugghe, Philippe G. Jorens

**Affiliations:** 1 Department of Anesthesia, Antwerp University Hospital, University of Antwerp, Wilrijkstraat 10, B-2650 Edegem, Belgium; 2 Department of Critical Care Medicine, Antwerp University Hospital, University of Antwerp, Wilrijkstraat 10, B-2650 Edegem, Belgium

**Keywords:** Clinical trials, Critical care, Outcomes, Mechanical ventilation

## Abstract

**Background:**

The red cell distribution width (RDW) is a widely available, inexpensive, and highly reproducible test that reflects the range of the red cell sizes. Any process that releases reticulocytes in the circulation will result in an increase in RDW. Elevated RDW values are linked to worsened pulmonary function in the adult population. We performed a retrospective cohort study to describe the association between RDW and respiratory failure in critically ill children in a in a pediatric intensive care unit (PICU) in a tertiary university hospital.

**Subjects:**

All patients admitted between January 2009 and June 2015 were considered eligible for inclusion.

**Methods:**

Retrospective cohort study.

**Results:**

In total, 960 patients were included in the cohort analysis. Of those patients, 149 (15.5%) had elevated RDW values. RDW on admission was associated with lower 28 day ventilator-free days. The highest quintile of RDW was associated with the need for mechanical ventilation, even when correcting for anaemia, age and Pediatric Risk of Mortality (PRISM) scores. In the subgroup of ventilated patients, RDW was associated with nadir PaO_2_/FiO_2_(P/F) ratios.

**Conclusion:**

The RDW value on admission of our PICU patients is associated with a greater need for invasive mechanical ventilation, lower 28 day ventilator-free days and lower nadir P/F ratios in the patients with highest RDW values on admission. RDW may be a valuable, cheap and universally available, prognostic parameter for respiratory dysfunction in the PICU.

## Background

The red cell distribution width (RDW) is routinely reported in automated complete blood counts. It is a widely available, inexpensive, and highly reproducible test reflecting the range of the size of the circulating red blood cells [1]. Any process that releases reticulocytes in the circulation will result in an increase in RDW [2].

More and more reports highlight that RDW may be indicative of a person’s underlying general health status, and more specifically reflects a certain degree of inflammation. The different factors that might increase the heterogeneity in size, such as the red blood cell circulation half-life and membrane deformability, can be influenced by inflammation too [3]. A correlation between RDW and C-reactive protein (CRP) values has been shown, and a higher RDW can be linked to increases in erythrocyte sedimentation rate and interleukin-6 levels as well [4].

RDW has been associated with adverse outcome and individual organ failure in both community-based cohorts and in critically ill adults [5–8]. Elevated RDW values have been found in adults with a wide variety of heart diseases, including stable coronary disease, acute myocardial infarction and pulmonary hypertension [9, 10]. Moreover, in the adult ICU population, RDW might be used as an independent predictor of mortality, and improve the current prognostic scores such as the SAPS and APACHE-II scores [8]. In the critical care unit, RDW is associated with higher mortality in adult patients with single organ failure i.e. acute kidney injury necessitating continuous renal replacement therapy [11]. RDW is linked in the general population with pulmonary dysfunction too [12].

As opposed to emerging studies in the adult (critical care) population, the value of RDW remains largely unexplored in the pediatric critical care population (pediatric critical care unit; PICU). We are aware of only two studies. In a report by Massin and co-workers, a high RDW value has been linked to worse clinical outcome in a cohort of children undergoing surgery for congenital heart disease [13]. Ramby and co-workers reported on the association between high RDW values on admission with worse clinical outcome in the PICU, and could predict it with comparable positive and negative predictive values as the commonly used disease severity scores like the Pediatric Index of Mortality (PIM) II score [14].

No studies on the association of organ dysfunction and elevated RDW values in the pediatric ICU population are thus far published to our best knowledge. Searching for prognostic factors for organ dysfunction in the PICU is however essential, as single and multiple organ dysfunction is the prime cause of death in the PICU population [15]. In this paper we present our findings of the associations of RDW with different outcome parameters reflecting respiratory insufficiency, including the need for mechanical ventilation, 28 day ventilator-free days and PaO_2_/FiO_2_ ratios in our cohort of children admitted to a tertiary PICU.

## Methods

### Study population

We retrospectively analysed all patients who were consecutively admitted to the 9-beds PICU between January 2009 and June 2015 of the Antwerp University Hospital, Edegem, Belgium. This PICU serves as a tertiary referral unit with a mixed medical and surgical population, but not for post-cardiosurgical patients or those in need for extracorporeal membrane oxygenation. All patients below 16 years old with an available baseline RDW value on admission to PICU were eligible for inclusion.

We excluded patients who were transferred to another intensive care facility and thus lost to follow-up, and those with ICU discharge within 24 h after admittance, those with hemoglobinopathies and bone marrow transplant patients, and those who received a blood transfusion prior to ICU admission.

The ethical committee of our institution granted permission for this trial (EC 11/02/19); a waiver of consent was granted as a retrospective review of existing data was performed and no intervention was/had been planned. The study was performed in accordance with the ethical standards of the Declaration of Helsinki.

### Patient data, outcomes and covariates

Demographic data, laboratory values, risk factors, and outcome parameters were extracted for review from the ICU Patient Data Management System (iMDsoft MetaVision). We calculated Pediatric Risk of Mortality (PRISM) III [16] scores as marker for disease severity for each patient using the available data. We defined invasive ventilation as being ventilated mechanically through an endotracheal tube or a tracheostomy. Patients who were ventilated on admission but who were subsequently extubated within 24 h after admission were categorized in the non-ventilated group, because these patients were in general patients that were quickly weaned after routine surgery. This was done to separate respiratory failure from temporary postoperative need for respiratory support. When a patient was re-intubated within 24 h after initial extubation, both episodes were considered as one (same case). Length of ventilation (LOV) is a commonly used clinical endpoint in lung disease studies. However, as many factors have an impact on the LOV [17] that may not be easily discerned in a retrospective analysis, we a priori decided to analyse LOV as a dichotomous outcome parameter. We chose 96 h as the cut-point for long-term ventilation, because of the use of this cut-point in previous research [17]. Patients who were ventilated for less than 24 h were not included in the length of ventilation analysis.

Our primary outcome parameter was the need for mechanical ventilation during the patients’ ICU stay. In the subgroup analysis of ventilated patients, we evaluated the association between RDW and nadir P/F values (= PaO2/FiO2 ratio as the ratio of arterial oxygen partial pressure to fractional inspired oxygen) during the entire ventilated period, 28 day ventilator-free days and length of mechanical ventilation of more than or under 96 h.

### Lab analysis

RDW was measured during the entire study period as part of the routine haematological tests using the Advia Hematology Analyzer (Siemens Healthcare Diagnostics, Deerfield, IL, USA) according to the formula: *RDW* = (*Coefficient of Variability of RBC* ÷ *mean MCV*) × 100. Periodic quality checks were performed as part of the clinical laboratory accreditation requirements. As RDW is associated with anaemia, we verified whether our patients were anaemic on admission. Reference values for RDW in our hospital are between 11.6% and 14.6% for the adult population, but evaluation of RDW abnormality was age-adjusted: when we considered the RDW as a dichotomous variable (normal/abnormal) we used the age-specific normal values [18].

### Statistical methods

Normality was assessed using the Kolmogorov-Smirnov test and results are reported as mean ± standard deviation (SD) for normally distributed or median (interquartile range) for non-normally distributed data. Patient characteristics between the ventilated and non-ventilated cohort were compared using Mann-Whitney U and Chi square tests. A linear regression model was used to assess the relation between RDW and hemoglobin levels on admission.

To test the association between RDW and the need for mechanical ventilation, the RDW values were split into quintiles. As a strategy to allow us to evaluate the RDW specific association with respiratory failure, we designed a regression model. We a priori decided to include the presence of anaemia on admission (with regards to the age of the patient), age, and the PRISM III score in the model, together with the RDW quintile. A logistic regression model was then constructed entering these data. A Kruskal-Wallis test was used to assess the association between 28 day ventilator-free days and RDW quintiles.

A subgroup analysis was then performed, entering the same variables in a linear regression model with the nadir P/F ratio and mechanical ventilation over or under 96 h as outcome parameters.

For all tests a *p* value less than 0.05 was considered statistically significant. All analyses were performed with SPSS v24 for Mac.

## Results

Over the 5-year period, we retrieved data from 1514 hospitalised patients. 394 patients were excluded because no RDW data from the admission to ICU date were available. 32 patients were excluded because they had hemoglobinopathies or were bone marrow transplant patients, and 128 patients were excluded because they had received a blood transfusion prior to ICU admission. In total, 960 patients were included in the cohort analysis. Of those patients, 149 (15.5%) had elevated RDW values. Median age was 3 years old (IQR 0–9.0), 56% were male, 68.2% were medical admissions. The median PRISM III score on admission was 10.0 (IQR 4.0–15.0). Table 1 shows the different demographic characteristics of our cohort, including the differences between the ventilated and non-ventilated subgroup.

**Table 1:** Population characteristics

	All patients	Ventilation subgroup	Mean difference (95% CI)	*P*
Patients included (*n*)	960	333		
Gender (male/female, %)	56/44	59/41		
Age (years)	3.0 (<1–9.0)	2.0 (<1–6.0)	−1.6 (−2.2 –0.9)	<0.001
Age group (*n*, %)				
< 1 year	259 (27.0)	112 (33.6)		
1–5 years	245 (25.5)	133 (40.0)		
6–12 years	334 (34.8)	54 (16.2)		
> 12 years	122 (12.7)	34 (10.2)		
Weight (kg)	14.0 (8.5–28.0)	12.0 (6.9–20.0)	–5.0 (−7.3–2.7)	<0.001
PRISM score	10.0 (4.0–15.0)	17.0 (13.0–23.0)	11.2 (10.4–12.1)	< 0.001
Reason for admission (%)				
Respiratory	25.0	30.3		
Trauma	9.9	9.9		
Postoperative	21.9	16.2		
Neurological	23.9	26.4		
Cardiac	3.2	5.7		
Renal	2.4	1.5		
Gastro-intestinal	2.1	0.9		
Intoxication	3.3	2.4		
Endocrinological	1.0	0.6		
Other	7.3	6.0		
Admission parameters				
Hb (mg/dL)	10.8 (9.5–12.1)	10.0 (9.0–11.4)	−0.8 (−1.1 −0.6)	< 0.001
Steroids (%)	15.2	17.1		0.23
Vasopressors/inotropics (%)	6.4	14.2		<0.001
Anaemia (%)	54.4	64.3		<0.001
CRP (mg/dL)	19.0 (4.0–67.4)	22.7 (7.9–77.5)	7.6 (−3.0–18.0)	0.16
WBC count (× 10^9^/L)	11.6 (8.1–16.1)	10.7 (7.2–15.9)	-0.6 (−1.5–0.35)	0.21
RDW on admission	13.8 (13.0–15.1)	14.2 (13.1–15.3)	0.4 (0.1–0.6)	0.002
ICU LOS (days)	2.5 (1.0–5.8)	6.6 (2.5–13.9)	8.9 (7.8–10.0)	< 0.001
ICU mortality (%)	4.3	10.2		< 0.001
RDW on admission quintiles (n, %)				
Quintile 1 (11.2–12.8)	181 (18.8)	49 (14.7)		
Quintile 2 (12.9–13.4)	209 (21.7)	65 (19.5)		
Quintile 3 (13.5–14.3)	198 (20.6)	66 (19.8)		
Quintile 4 (14.4–15.4)	189 (19.7)	78 (23.4)		
Quintile 5 (15.5–29.6)	183 (19.1)	75 (22.5)		
28 day VFD		23.0 (15.0–26.0)		
Nadir P/F ratio during MV		112 (72–231)		

Data as median (IQR) unless specified otherwise

*PRISM* Pediatric Risk of Mortality, *Hb* haemoglobin, RDW Red cell Distribution Width, *ICU* Intensive Care Unit, *MV* mechanical ventilation, *P/F* PaO2 / FiO2, *28 day VFD* 28 day ventilator-free days, *LOS *length of stay. Mean difference versus non-ventilated subgroup (data not shown)

The ventilated subgroup was generally more ill, as can be expected. The median RDW on admission for all patients was 13.8% (IQR 13.0–15.1%). Median RDW for the third day after admission was 14.4% (IQR 13.4–15.6%) and day eight was 15.0% (IQR 13.9–16.5%). A postoperative status was not associated with RDW values on admission.

RDW on admission quintiles were inversely associated with 28 day ventilator-free days (*P* = 0.003). The proportion of patients with the necessity to be invasively ventilated increased in a step-wise fashion for each successive RDW group (Table 2).

**Table 2:** Ventilation parameters for each Red cell Distribution Width quintile

RDW quintile	Need for MV (%)	MV >96 h in ventilated subgroup (%)
Quintile 1	27.1	52.3
Quintile 2	31.1	45.0
Quintile 3	33.3	56.1
Quintile 4	41.3	57.0
Quintile 5	41.0	68.6

*RDW* Red cell Distribution Width, *MV* Mechanical ventilation

In a logistic univariate regression model, RDW quintile was significantly associated with the need for mechanical ventilation (*P* = 0.021). In the multivariate regression model, anaemia and PRISM III scores on admission remained significantly associated with the need for invasive mechanical ventilation, but RDW quintile as a parameter did not. However, the fifth RDW quintile had an OR 2.6; 95%CI 1.4–4.9; *P* = 0.004 for mechanical ventilation. Table 3 summarizes this regression model.

**Table 3:** Uni- and multivariate regression model predicting the need for MV

Parameter	P (univariate)	Exp(B)	P (multivariate)	95%CI
RDW quintiles	0.021		0.065	
RDW quintile (2)	0.047	1.48	0.287	0.72–3.07
RDW quintile (3)	0.160	1.66	0.152	0.83–3.30
RDW quintile (4)	0.549	1.40	0.326	0.72–2.73
RDW quintile (5)	0.018	2.59	0.004	1.36–4.95
Age	<0.001	0.96	0.096	0.92–1.01
Anaemia	<0.001	2.59	0.007	1.30–5.16
PRISM III	<0.001	1.32	<0.001	1.24–1.41

*RDW* Red cell Distribution Width, *MV* Mechanical ventilation

**Fig. 1:**
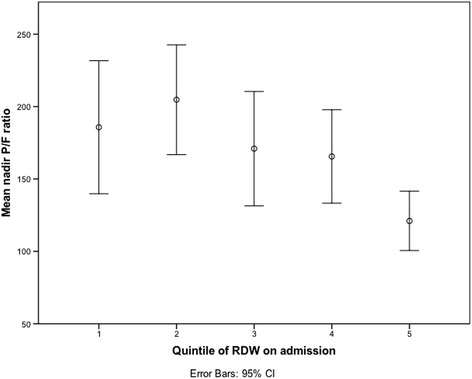
mean nadir PaO2/FiO2 (P/F) ratio for each red cell distribution width (RDW) quintile

In the subgroup analysis of ventilated patients, RDW was associated with nadir P/F ratios (*P* = 0.003) in a linear regression model with anaemia (NS) and PRISM III scores (*P* < 0.001) included in the model. Fig. 1 displays the association between the quintile of RDW on admission and nadir P/F ratios. We did not find a statistically significant relation between the need for MV > 96 h and the RDW quintile on admission.

These regression models demonstrate the association of RDW values on admission with respiratory failure in our pediatric cohort, even when correcting for the confounding factors anaemia, age, and disease severity (PRISM III).

## Discussion

Our data demonstrate that RDW at the time of PICU admission is associated with different validated parameters for respiratory failure in our cohort of PICU patients. The need for mechanical ventilation, and the nadir P/F ratios in ventilated patients are both associated with RDW values on admission, and largely remain so in regression models after correcting for both disease severity and anaemia.

Why is this relevant? An increase in RDW can be linked to hypoxemia [2]. Transient decreases in oxygen partial pressures (PaO_2_) will lead to a ‘pulsed’ erythropoietin (EPO) release through hypoxia-inducible transcription factors. These will in its turn trigger the release of immature reticulocytes into the circulation leading to anisocytosis and a higher RDW in the affected patient [2]. Supporting this hypoxemia – anisocytosis pathway and the value of RDW in lung pathology, elevated RDW values have been found in diverse respiratory disease processes reflecting differences in disease severity. Grant has linked higher RDW values with worse pulmonary function tests in a cohort of patients without clinically evident respiratory diseases [12]. Similarly, Sincer has demonstrated a higher RDW in patients with COPD compared to a control group, even when corrected for known reasons for a high RDW such as folate, iron or vitamin B12 deficiencies [19]. The association between RDW and mortality has been shown in COPD patients [20]. Furthermore, in a cohort of patients with pulmonary embolism, an elevated RDW on admission was associated with worse hemodynamic parameters and early mortality [21]. Also, higher RDW values could be linked to worse long-term outcome after pulmonary embolism, with a higher percentage of late mortality and post-pulmonary embolism pulmonary hypertension in patients with a high RDW [22]. Until recently, the value of RDW in children admitted to the PICU had not been reported. A recent study by Said et al. in critically ill children showed that admission RDW is associated with pediatric ICU mortality and morbidity, independent of illness severity [23]. The relationship between RDW and organ failure was not studied specifically, although there was a weak association between the RDW value and the number of ventilation free days [23]. The observed worse respiratory outcome for patients with a higher RDW may have several underlying reasons. The independent association of RDW with outcome may imply that anisocytosis itself may be a possible causable factor in organ dysfunction. The role of the microcirculation has been suggested in this pathophysiological process [24, 25]. In a recent study, Fontana et al. discuss the relationship between RDW values and microcirculatory changes using sublingual Sidestream Dark Field videomicroscopy in a cohort of patients with sepsis [26]. The RDW was elevated in 61% (*n* = 74) patients in this study, and the authors found no correlation between RDW and the microcirculatory parameters. This shows that RDW reflects a general degree of illness in a patient, and emphasizes the added value of RDW in evaluating a patient’s general condition. The median RDW in their cohort was 13.8%, more than their upper-normal limit of RDW of 13.4%, indicating the worsened physical condition in this group. However, as not all septic patients had elevated RDW values, it becomes clear that the RDW is not a specific marker of this disease. Furthermore, the lack of relation between the RDW and microcirculatory parameters is interesting, as we might assume that an increase in RDW would result in more abberant and large erythrocytes that could obstruct the capillaries. This anisocytosis and its role in the complex field of microcirculation in different organs in the critically ill patient warrants further research. Other factors may be involved as well. Firstly, the presence of anaemia is the most likely cause of a change in RDW, which is the reason we corrected our statistical model for this parameter [24]. Secondly, oxidative stress, present in lung injury and ventilated patients through the generation of reactive oxygen species shortens the RBC lifespan [3]. Oxidative stress will thus promote the release of young cellular forms into the circulation. Thirdly, another factor potentially causing a change in RDW is vitamin D3 deficiency [27]. Vitamin D3 plays an important role in erythropoiesis and cell proliferation, so a small change in vitamin D3 concentration will affect bone marrow erythropoiesis. But more generally, it has also been proposed that RDW may reflect the patient’s degree of physiologic reserve or chronic inflammation [8]. This hypothesis is further supported by the fact that a RBC’s half-life is approximately 28 days, meaning that RDW on admission to the PICU was influenced by a chronic state or an event that happened some time before this measurement was made.

Many confounding factors should be taken into account when considering the prognosis of patient’s disease, e.g. the underlying disease and inflammatory markers like CRP, LDH, or lactate levels. RDW is possibly influenced or associated with these factors. Regardless of these possible influencing factors, RDW is attractive as a pragmatic clinical biomarker for respiratory dysfunction by its low cost and universal availability compared to other proposed biomarkers. Interestingly, the CRP level and WBC count level on admission were not associated with the need for mechanical ventilation, in contrast to RDW and RDW quintiles.

Despite the robust data, our study has several limitations. Since this was a secondary analysis of an existing database, we were not able to assess all possible confounding variables, such as measures of iron deficiency, markers of nutritional status, or other biomarkers of inflammation. Our cohort was too small to assess whether the PRISM and PIM scores may be improved in their possibility to predict adverse outcome in PICU patients when incorporating RDW values in their score calculation, but this has been a topic of recent research [8]. We did use RDW values on admission, but it is possible that the values were already increased during the days preceding the admission. This study does not provide an answer as to whether an increase of RDW is a consequence of, or a reason for respiratory dysfunction. It is already shown that inflammation increases RDW values, and inflammatory pathways will cause worse pulmonary function. Criteria for sepsis were not routinely recorded on admission for our patients. This limits us in interpreting the results, as we are unable to take the context of sepsis into account when assessing RDW as an independent biomarker for respiratory dysfunction. Neverteless, as previously mentioned, sepsis is not always associated with increased RDW values [26]. The confounding factor of age is to be taken into account as well. Younger patients admitted to the ICU are already at higher risk for organ dysfunction, have physiologically higher RDW values, and are thus over-represented in the higher RDW quintiles. We thus decided to include age as a confounder in the multivariate analysis. We did not record reasons for tracheal intubation, and thus cannot differentiate between patients that received mechanical ventilation because of low oxygenation, insufficient work of breathing (i.e. insufficient ventilation) or a combination of these. We used P/F ratios as a parameter for oxygenation dysfunction. This parameter, although still widely used in clinical practice and clinical trials, ignores the effect of the mean airway pressure (Paw) on oxygenation. As an alternative parameter, the oxygenation index (FiO_2_ × Paw/PaO_2_) takes the Paw into account and is by many considered to be a better indicator of lung injury. Unfortunately, the Paw was not available for a substantial amount of patients in our database, and for this reason we were unable to include the oxygenation index as a parameter for oxygenation dysfunction***.***

In summary, the RDW value on admission of our PICU patients was independently associated with respiratory failure, reflected by the greater need for mechanical ventilation, fewer ventilator-free days and lower nadir P/F ratios in the patients with highest RDW values on admission. As a widely available and pragmatic biomarker, the RDW value is able to predict who is at risk for respiratory failure in our cohort, allowing for an early tailored approach toward respiratory monitoring. RDW may be a valuable prognostic parameter for lung or other organ dysfunction in the PICU. Further research is needed to confirm this link for the different organ systems in pediatric populations. Future studies in the field of microcirculation may have their value in assessing the cause-effect relation of organ dysfunction and anisocytosis in critically ill children.
